# A behavioral dataset of predictive decisions given trends in information across adulthood

**DOI:** 10.1016/j.dib.2024.110832

**Published:** 2024-08-10

**Authors:** Daniel Sazhin, Vishnu Murty, Chelsea Helion, David V. Smith

**Affiliations:** Temple University, United States

**Keywords:** Strategic, Dynamic, Trends, Function learning, Cognition

## Abstract

Making early and good predictions is a critical feature of decision making in domains such as investing and predicting the spread of diseases. Past literature indicates that people use recent and longer-term trends to extrapolate future outcomes. Nonetheless, less is known about what differentiates the strategies people use to make better predictions than others. Furthermore, factors underlying predictive judgments could be an important behavioral component in psychosocial research investigating manic-depression, anxiety, and age effects. Additionally, predictive judgments may be moderated based on the experience of living in areas with greater income inequality. To address these issues, we used investment tasks where participants had to predict future outcomes of their investments based on a trend in information. In the task, participants predicted how many tokens a gold mine would produce on the twelfth turn. On each turn, participants could ask for more information at a cost, or make a prediction about whether the gold mine would produce more or less than 100 tokens by the 12th turn. The trend was determined by function type (exponential and inverse exponential functions), whether the function was more linear or curved (growth factors), and good or bad outcomes (final values). This paradigm could help disentangle to what degree people use recent or longer-term information to inform their predictive judgments. We used Qualtrics to conduct this study. We also collected questionnaire data quantifying anxiety, impulsivity, risk attitudes, manic-depressive symptoms, and other psychosocial characteristics. The study was administered to adults with age ranges across the lifespan (*N* = 360; 225 male, 132 female; 3 nonbinary; mean age: 44.3 years; SD: 15.4 years, min: 18 years, max: 78 years). Additionally, we sampled across areas with high- and low-income inequality, thereby allowing researchers to investigate if value-based decisions are associated with participants’ local communities. We outline potential ways to use and reuse this data, including exploring how individual differences are associated with predictive judgments.

Specifications Table

**Table:** 

Subject	Psychology: Experimental and Cognitive Psychology
Specific subject area	Function learning and predictive judgments
Type of data	experimental design information (tab-separated values [.tsv]) experiment meta-data (Java Script objection notation [.json])
Data collection	Data was collected online using a targeted Qualtrics panel. We sampled across education demographics (40 % without college degree), age (21–90), and zip codes with greater income inequality.
Data source location	Institution: Temple University Location: Philadelphia, Pennsylvania, USA
Data accessibility	Repository name: Github Data identification number: https://zenodo.org/doi/10.5281/zenodo.11513694 Source code: https://github.com/DVS-Lab/StayGo2-DataInBrief/

## 1 Value of the Data

• Making accurate predictions is important and challenging in many real-world contexts that change over time, such as in investing, medicine, the military, and aviation. Making poor predictions can result in major consequences, such as low returns for individual investors on the stock market [1] or delayed responses to an epidemic [2]. While advances in cognitive science and computational modeling have revealed important components of predictive judgments, it remains unknown what differentiates the strategies people employ to make better predictions than others. This study helps address how people make predictions.
• This behavioral data is valuable for understanding how people make predictive judgments based on trends in information. While past investigations have suggested that people value both recent and average trends [[3], [4], [5]] when making predictions and that people explore more with uncertain information [[6], [7], [8]], it remains unclear how people incorporate these components when predicting the outcomes of future trends. Additionally, it is unclear how people explore information when making longer-term predictions. Our design controlled for outcomes across trends and assigned a cost to obtaining information.
• The task consisted of several conditions to assess how people make predictive judgments. Each task varied the difficulty of prediction through flatter or steeper curves and with exponential versus inverse exponential information (e.g., curves that start slow and speed up versus ones that start fast and slow down). Half of the curves ended below a threshold of 100 and the other half above 100, and the participant was tasked with predicting this outcome. Participants decided how many turns (up to six) to track the trend before making their prediction. This design helps understand how function types, steepness of the curve, or the trend outcomes affected predictions, and how long people took to make their choices and their general predictive efficacy. Overall, this approach can help determine if people track longer-term trends, recent information, and how trends affect how people incorporate these components into their predictive judgments.
• This data can be reused by other researchers to assess the relations between predictive judgments and psychosocial factors measured. For example, Major Depressive Disorder (MDD) may incorporate inaccurate extrapolation of future outcomes, yielding inaccurate beliefs that compound disordered thinking due to abnormalities in reward processing [9], potentially interfering with valuation processes in predictive judgments. Specifically, we include measures of manic-depressive symptoms (7Up and 7Down), risk attitudes (DOSPERT), anxiety (PROMIS), impulsivity (ABIS), cognitive decline (ECog), socioeconomic status, and loneliness. Additionally, latent variables such as response times can be assessed to understand variability in predictive judgments. Response times can be applied to modeling decision dynamics (e.g., drift diffusion modeling; [10] in predictive judgments, revealing for example if people accumulate information differently over higher turns before they make their decision.
• Additionally, it is possible to assess how sociodemographic and geographic individual difference measures are related to value-based decision making. We collected geographic data across the sample, assessing areas with high and low-income inequality [11]. Thus, it is possible to investigate if participants’ value-based choices are associated with attitudes toward gambling and investing, and if their risk attitudes or decisions are moderated by living in areas with high or low income inequality.

## 2 Background

Making accurate predictions is important, yet it remains unknown what differentiates how people make better predictions than others. To understand how people make predictions, researchers investigate how people learn in uncertain situations over time [[12], [13], [14]]. Dynamic and uncertain environments are often viewed through the lens of exploiting resources or exploring alternatives with higher payoffs [15]. Thus, the decision to stay or go in explore-exploit tasks is usually informed by the last turn of the slot machine [14] or the foraging patch output [16], and this is a nearly perfect predictor of the expected output next turn. Nonetheless, predictive judgments require considering future time points beyond the next turn [[3], [4], [5]]. Some people may prefer to use a strategy that focuses on recent changes in information whereas others may use a more rules-based approach and extrapolate average trends over time [17,18] when making their predictions. Overall, assessing how people adapt their predictive judgments can be accomplished through studying how changes in subjective beliefs of future outcomes change over time. Additionally, it remains unknown how psychosocial factors may affect predictive judgments. Understanding the mechanisms underlying predictive judgments could help develop interventions aimed at improving decision making.

## 3 Data Description

The data are available on GitHub, and a permanent snapshot of data versions is available on Zenodo [19]. The dataset is composed of behavioral data from 360 participants who completed a predictive decision-making task. Participants played a game where they were told how many tokens were coming from a gold mine, with a goal of identifying whether the mine would output more or less than 100 tokens at a future time. The task stimuli and survey questions are available for researchers to review in the “survey_questions.docx” file within the GitHub “code” directory. Additionally, the survey is reproducible by accessing the .qsf file located in the code directory and uploading it into Qualtrics.

The data are organized in accordance with the Brain Imaging Data Structure (BIDS) specification [20]. The BIDS format for organizing data is used extensively to report behavioral and neuroimaging data in cognitive neuroscience as it is standardized, thereby making data easier to work with and reproducible. Extensive online documentation of the files and format is available online and the data could potentially be accessed by external data analysis software packages that use BIDS formatting. As part of the BIDS standard, files use their suggested directory structure and the data is accessible using GitHub. We include README files in each folder to guide researchers to the contents of each folder, though we briefly describe the database structure. The scripts used to extract the behavioral and survey data are in the code directory. Within the bids directory, we include the sourcedata, which is the raw participant data, and each subject's behavioral data within a labeled participant folder. Also, we include a phenotype folder with the corresponding survey data administered in the study. Consistent with BIDS formatting, we include .json sidecars that contain metadata regarding the behavioral and survey files. All of the behavioral and survey data are formatted in TSV format and can be easily accessed by any statistical programming software (e.g., R, MATLAB, Python) for future analysis.

**Table:** 

Data Files	Description
participants.tsv	Basic demographics for each participant (education level, occupational prestige, age, gender, race, ethnicity, zip code, when they moved and SES information, and level of income inequality within the zip code.
sub-*/beh/sub-*_task-staygo2_beh.tsv	Eleven column file describing the block order, trial type, decision phase, turn stayed or left, tokens displayed, growth factor, final value, correct/incorrect, stay/eft, earnings, response time and onset time.
sub-*/beh/ sub-*_task-*_ staygo2comps_beh.tsv	Seven column file describing the four comprehension check questions administered after the task portion. Participants specified predictions on four trends (C1 through C4) on Day 7 and Day 12 respectively.
phenotypes/*	Exported questionnaire data. We provide each subject number and each question serves as a column. We report the abbreviated impulsiveness scale, DOSPERT, EcoG, Loneliness, PROMIS, and SevenUp-SevenDown questionnaires.

## 4 Experimental Design, Materials and Methods

### 4.1 Participants

We recruited a total of 360 participants across the United States (225 male, 132 female; 3 nonbinary). We used an online Qualtrics panel to perform the Gold Mine task for this study. All participants gave informed consent in accordance with the Institutional Review Board at Temple University. Participants were paid by Qualtrics for completion of the survey. The task was incentive compatible as participants were instructed that they might be selected for bonus payout based on a single decision in the task. We used a debrief form to indicate that the bonus payment was a randomly selected fixed $25 bonus. We used a fixed bonus instead of an actual decision in order to ensure that all participant choices remained deanonymized. Due to issues in task implementation, we used the soft launch period where 10 % of the initial data were used (*N* = 36) to pilot the study and make adjustments to the instructions and attention checks to ensure comprehension. Thus, while we include *N* = 360 in the dataset, we recommend using participants starting at subject 37. Nonetheless, across all 360 participants, the mean age was 44.3 (minimum: 18; maximum: 78; SD: 15.4 years) and was mostly not Hispanic (289 participants); (248 white, 12 Asian, 60 Hispanic/Latino, 13 —Two or more races, 27 other). 154 participants had a college degree or greater, leaving 206 participants with less than a college degree. Participants were sampled across areas with greater or lower income inequality [11]. Through parameterizing alpha and gamma values of the GINI coefficient we identified 50 counties with the highest and lowest gamma and alpha values respectively. We found the corresponding zip codes within these counties (*N* = 818 zip codes) to target participants. The list of zip codes used for targeting is located in the code directory, in the “ortega_parameters_all_zipcodes.csv” file. Participant demographic data, including age, race, gender, ethnicity, education, zip code, and social economic status, are located within the “participants.tsv” file within the BIDS directory.

### 4.2 Task parameterization

**Fig. 1:**
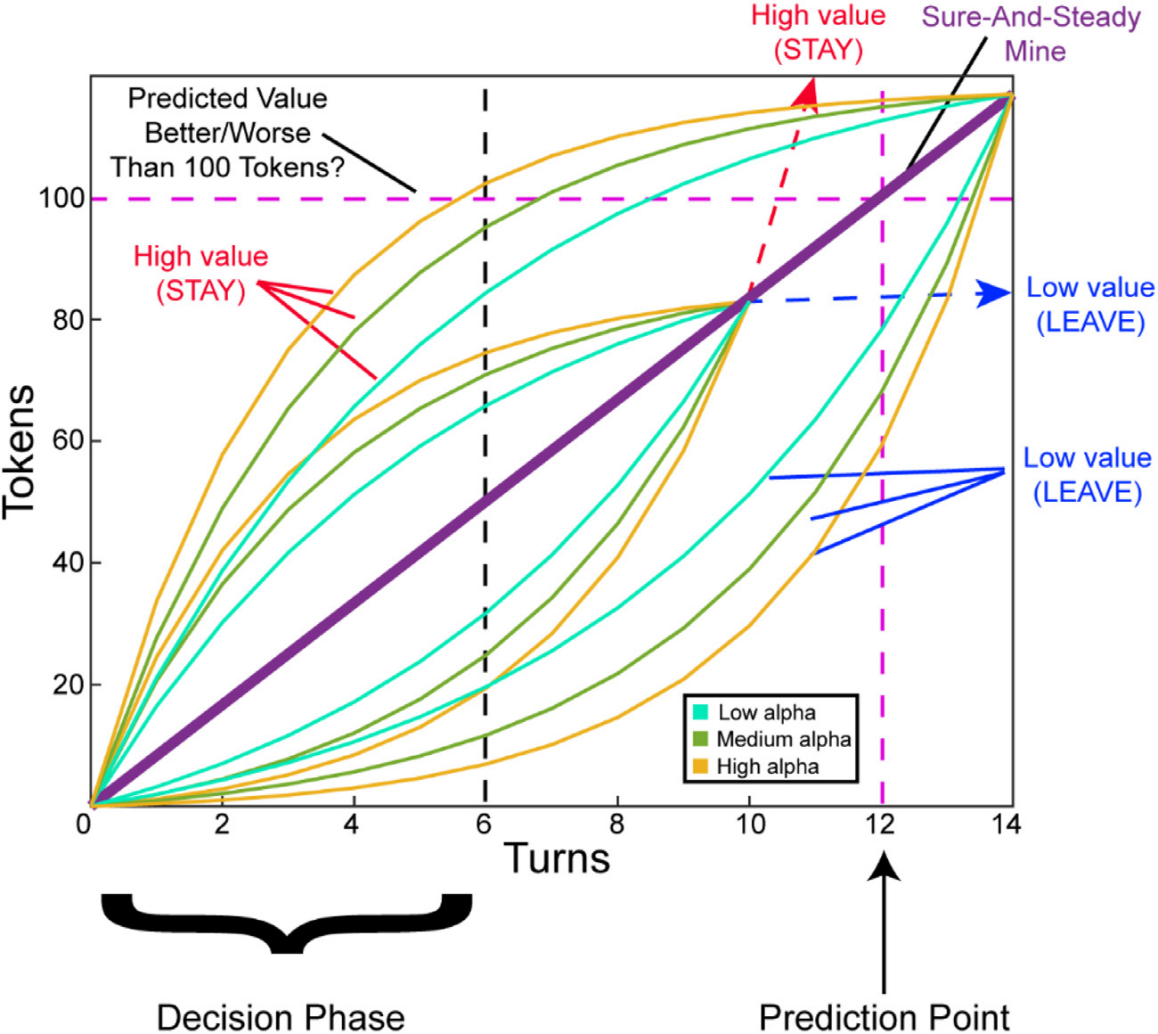
Parameterization of exponential and inverse exponential trends. Participants experienced exponential and inverse exponential trends before they predicted future outcomes. Half of the mines had high value outcomes (above 100 tokens) and the other half had lower value outcomes. Some mines had flatter/ more linear curves (low alpha) and other mines had steeper curves (high alpha). Participants experienced medium alpha curves during practice and comprehension checks. Participants had six turns to decide whether they want to stay with their gold mine or to leave to a sure-and-steady mine that made 100 tokens for sure by the 12th turn. Participants should select STAY for high-value mines and LEAVE for low-value mines.

A trial consisted of 6 decision turns, where a participant selected whether to get more information about their trend by advancing to the next turn, or to make a decision to stay or leave their gold mine. Participants experienced both exponential and inverse exponential functions. Within each sample, participants experienced 2 growth factors and 2 final values, and the rounds repeated 3 times, yielding 24 rounds per sample. The growth factors included alphas of 1.2 and 1.4. The final values were 83 and 117 tokens, which constrained the respective functions to cross the alternative patch at the 10th and 14th future turn respectively. These parameters were fixed across exponential and inverse exponential functions (see Fig. 1). The practice trials and comprehension checks consisted of 4 trials with an alpha of 1.3 and final values of 83 and 117 respectively.

### 4.3 Task design and procedure

**Fig. 2:**
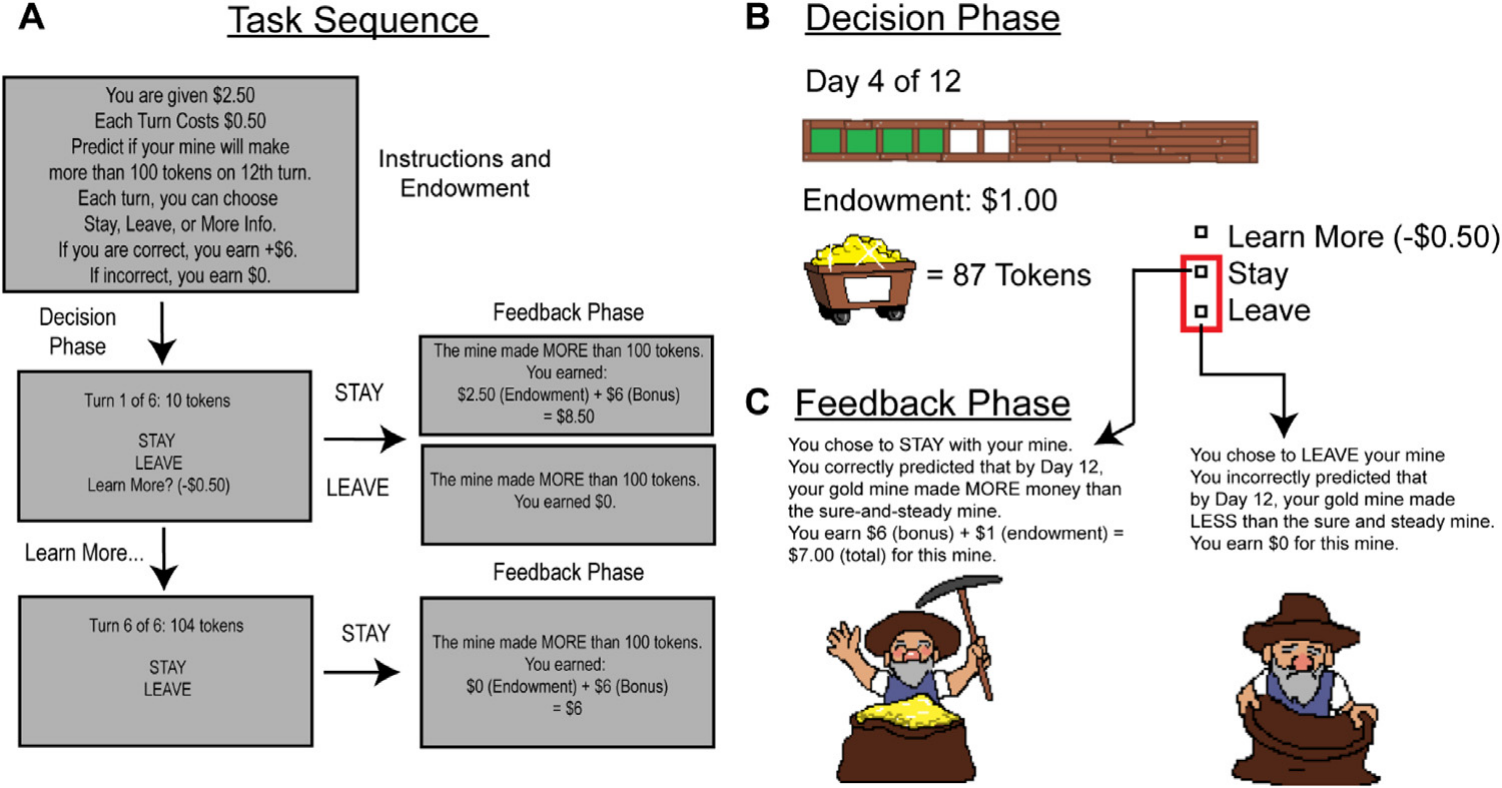
Gold Mine Task. The Gold Mine task presents a trinary choice (More, Less or More Info) to participants. The goal of the game is for participants to decide if a gold mine will produce more or less than 100 tokens on the 12th turn. Each turn costs $0.50, thus the participant is motivated to make a prediction as soon as possible. Participants can pay for more information or make a prediction sooner. In Panel A, we describe the instructions and endowment received, along with the task sequence. Participants have six turns to observe the trend in information. In Panel B, we show an example of the stimuli participants received on one of the turns. Participants observed a progress bar that indicated how many turns left (of six) they could track information, along with how much endowment was left and how many tokens their mine output this turn. In Panel C, we indicate how participants experienced the Feedback Phase. In this example, the correct decision was to Stay, and the participant would have earned $7.00. If they selected Leave, they would have earned $0.

A participant received a cover story that they would predict the output of the mine. Some mines initially extracted less tokens than others but sped up over time. Others slowed down in their output over time. Nonetheless, the goal was to identify whether at the 12th turn their mine would have produced more or less than 100 tokens. Half of the mines produced less than 100 tokens. In the beginning of a round, the participant was endowed with $3 and had up to 6 turns to make their prediction (see Fig. 2). On each turn, they selected STAY, LEAVE, or More Information. If they chose More Information, they paid $0.50 to advance one more turn to see how much gold the mine made on the next turn. If they chose STAY or LEAVE, the round ended and they saw if their prediction was correct. If they were correct, they earned an additional $6 above their initial endowment. If they were incorrect, they earned $0 for that round. Participants were told that to earn the most money, they should decide as soon as possible whether to STAY or LEAVE their mine while still making correct choices. After receiving the instructions, participants experienced two practice rounds (randomly selected that is a High or Low outcome) and began the 24 rounds of the task.

After the task portion, we incorporated an accuracy/comprehension check to ensure that participants were making predictive judgments. After the last trial of the experiment, participants experienced four blocks where they simply observed the trend in tokens. Then they were asked to specify the 7th and 12th turn token outcomes. Additionally, we asked participants if they would have left or stayed with their mine. These reported results could be used as an exclusion criteria, as participants who consistently failed to stay with their mine if they received over 100 tokens did not understand the task. After the comprehension checks were completed, participants completed several surveys.

### 4.4 Questionnaires

We collected self-reported data on risk taking attitudes, loneliness, manic-depressive symptoms, impulsivity, anxiety, and self-reported cognitive decline. These measures are stored in the phenotype directory in the BIDS dataset. The accompanying JSON sidecar file contains all of the necessary metadata to reconstruct the items in each scale and the original citations for the measures. We measured the reliability coefficient of each measure using McDonald's Omega.

*Manic-Depressive Disorders:* To measure mood symptoms, we incorporated the 7Up7Down Scale (7U7D). This 14-item survey measure is divided into two scales measuring manic (7up) and depressive (7down) symptoms. The answers were coded on a four level Likert scale ranging from Never or Hardly Ever to Sometimes, Often, and Very Often or Almost Constantly. One example of a question includes, “Have you had periods of extreme happiness and intense energy lasting several days or more when you also felt much more anxious or tense (jittery, nervous. uptight) than usual (other than related to the menstrual cycle)?” All items were cumulatively coded across the two subscales to measure the level of manic and depressive symptoms in our sample of participants. The manic symptoms subscale (7Up) had good internal reliability (ω = 0.89), along with the depressive symptoms subscale (7Down) (ω = 0.91).

*Cognitive Ability*: To assess how everyday cognition affected predictive judgments, we used the Everyday Cognition Scale (ECog). The ECog survey is a 12-item short-form questionnaire that assesses cognitive decline. The questions use a five item Likert scale ranging from Better or No Change to Much Worse, with questions addressing memory, understanding directions, thinking ahead, and the ability to multitask. One example of a question includes rating one's ability to be, “Cooking or working and talking at the same time.” This questionnaire is a self-report of cognitive abilities compared to a 10-year-old, thereby reflecting cognitive ability or decline. The questionnaire score was averaged across all questions, minus questions where the participant selected “Don't Know” (coded as 999), thereby providing an average score ranging from 1 to 4 and incorporating only responses that were answered by the participant. The measure had good internal reliability (ω = 0.72).

*Risk Assessment:* To measure how risk attitudes in various types of risk-taking domains may be associated with predictive judgments, we used the Domain Specific Risk-Taking (DOSPERT) scale. This questionnaire assessed the degree to which the participant takes risks in domains including gambling, investing, health/safety, recreational, ethical, and social decisions. One example includes the participant rating the likelihood of them, “bungee jumping off a tall bridge.” Each domain included five questions, with a total of 30-items across the full questionnaire. The questionnaire employs a 7-item Likert scale ranging from Extremely Unlikely to Extremely Likely. The questionnaire was scored cumulatively within each domain and also across the entire survey (ω = 0.92).

*Impulsivity:* To measure if impulsivity was related to predictive decision making, we used the 11-item Abbreviated Impulsiveness Scale (ABIS)**.** This scale incorporates subscales for motor, non-planning, and attentional impulsivity, including questions such as “I do things without thinking.” The scale employs a 4-item Likert scale ranging from Rarely to Always, with questions addressing the degree to which people are future oriented, their perceived self-control, and planfulness. The measure was scored cumulatively across the three subscales, with a good reliability coefficient (ω=0.89).

*Anxiety:* The Patient-Reported Outcomes Measurement Information System (PROMIS) questionnaire is a 7-item subscale that assesses levels of anxiety, with questions such as, “I felt fearful.” Overall, the survey assessed a participant's self-reported feelings of how fearful, anxious, worried, nervous, tense, and they felt over the past seven days. The questions used a 5-point Likert scale ranging from Never to Always and were scored cumulatively and had good internal reliability (ω = 0.79).

*Loneliness:* The Loneliness questionnaire is a 3-item measure that assesses self-reported loneliness. The questions use a 3-point Likert scale ranging from Hardly Ever to Often. The questions assess relational connectedness, social connectedness, and self-perceived isolation. One example of a question includes, “How often do you feel isolated from others?” The questionnaire was scored cumulatively and had good internal reliability (ω = 0.86).

**Table:** 

File name	Goal of measure
abbreviated_impulsiveness_scale.tsv	Measures impulsivity by addressing the degree to which people are future oriented, their perceived self-control, and planfulness.
domain_specific_risk_taking_scale.tsv	Measure risk taking in domains including gambling, investing, health/safety, recreational, ethical, and social decisions.
everyday_cognition_questionnaire.tsv	Measures cognitive decline by assessing memory, understanding directions, thinking ahead, and the ability to multitask.
loneliness_questionnaire.tsv	Measures self-reported loneliness.
promis_questionnaire.tsv	Measures self-reported anxiety by assessing how fearful, anxious, worried, nervous, tense, and uneasy a person feels.
sevenup_sevendown_questionnaire.tsv	Measures manic and depressive tendencies

## Limitations

This dataset has four noteworthy limitations. First, due to errors in data collection, the dataset is missing feedback from four outcomes. In one instance, the feedback was presented, though the response time was not recorded. In the other three instances, the feedback phase was missing entirely. A second limitation is that 21 % of the participants failed to be manipulated to track the trend in information since they chose to always Stay or Leave their mine on the first turn rather than selecting Learn More to collect more information on subsequent turns. While this limits the power to assess psychosocial factors related to predictive judgments, it may offer the ability to contrast predictive judgments with gambling decisions. A third limitation is that sampling was limited to 24 rounds of the game following a short practice period. Predictive judgments may potentially have been improved with more trials across multiple parameters following a longer practice period. A fourth limitation is that the data was collected online and lacked experimental control available when administering experiments in a lab. Nonetheless, Qualtrics includes extensive quality control and attention checks to ensure that participants are responding satisfactorily. Qualtrics excludes participants that respond abnormally fast or slow, tracks for bot responses, and includes attention checks. Additionally, we included a “commitment to quality” question early in the survey. Finally, while none of the data provided by Qualtrics was excluded, we note that researchers can use the “comprehension check” portion of the behavioral experiment to exclude participants that performed less adequately in the task

## Ethics Statement

The study provided an informed consent and debrief form to engage in the study and share their de-identified data publicly. The study was approved by the Institutional Review Board at Temple University (Philadelphia, Pennsylvania, USA) under Protocol Number 28455, and it was conducted in accordance with the Declaration of Helsinki.

## CRediT authorship contribution statement

**Daniel Sazhin:** Conceptualization, Methodology, Software, Formal analysis, Investigation, Data curation, Writing – original draft, Writing – review & editing, Project administration, Visualization. **Vishnu Murty:** Conceptualization, Methodology, Writing – original draft, Writing – review & editing. **Chelsea Helion:** Conceptualization, Methodology, Writing – original draft, Writing – review & editing. **David V. Smith:** Conceptualization, Methodology, Software, Formal analysis, Data curation, Writing – original draft, Writing – review & editing, Supervision, Project administration, Funding acquisition.

## Data Availability

A behavioral dataset of predictive decisions given trends in information across adulthood (Original data) (Github).
